# Use of Limiting Dilution Method for Isolation of Nucleus Pulposus Mesenchymal Stem/Progenitor Cells and Effects of Plating Density on Biological Characteristics and Plasticity

**DOI:** 10.1155/2017/9765843

**Published:** 2017-10-08

**Authors:** Linghan Lin, Zhiwei Jia, Yachao Zhao, Yaohong Wu, Xiyan Zhao, Yong Li, Ziming Guo, Jiahai Chen, Shi Cheng, Deli Wang, Dike Ruan

**Affiliations:** ^1^Department of Orthopaedics, Navy General Hospital, Beijing, China; ^2^The Third Clinical College, Southern Medical University, Guangzhou, China; ^3^Department of Orthopaedics, The 306th Hospital of People's Liberation Army, Beijing, China; ^4^Guang'anmen Hospital, China Academy of Chinese Medical Sciences, Beijing, China

## Abstract

**Objectives:**

To evaluate the effects of the limiting dilution method and plating density in rat nucleus pulposus mesenchymal stem/progenitor cells (NPMSCs).

**Materials and Methods:**

Nucleus pulposus tissues were isolated from 12-week-old male Sprague-Dawley rats and NPMSCs were isolated using limiting dilution method. Cells were then classified into 3 groups according to plating density. Cell morphologies were observed, and colony-forming units, migration abilities, proliferative capacities, cell cycle percentages, multilineage differentiation capacities, stem cell biomarker expression levels, and immunophenotyping were also examined in each group.

**Results:**

Low density group (LD) had higher morphological homogeneity, stronger colony-forming ability, higher cell proliferation capacity, and enhanced cell migration ability relative to the other two groups (*p* < 0.05). Moreover, LD had more cells entering S phase, with fewer cells arrested in G0/G1 phase (*p* < 0.05). While all three density groups showed a multilineage differentiation potential, LD showed a higher degree of observed and semiquantified lineage specific staining (*p* < 0.05). Furthermore, LD displayed higher expression levels of stem cell biomarkers (Nanog, Oct4, and Sox2) and showed higher percentages of CD29+, CD44+, and CD90+ cells (*p* < 0.05) following flow cytometry analysis.

**Conclusions:**

Limiting dilution method is suggested when isolating NPMSCs as a means of improving cell activity and plasticity.

## 1. Introduction

Lower back pain (LBP) is a public health problem, with estimated annual health care system costs exceeding $100 billion in the United States [1, 2]. And intervertebral disc degeneration (IVDD) is one of the main causes of LBP [3]. Despite the numbers of affected individuals, the pathogenic mechanisms contributing to IVDD remain poorly understood. In recent years, endogenous stem cells have been isolated from almost all tissues and organs of the body. Stem cells play a key role in maintaining homeostasis and are now believed to serve as important disease-causing determinants [4]. Hence, mesenchymal stem cells (MSCs) implantation has become a promising method for the treatment of IVDD [5].

Intervertebral disc (IVD) tissue, which is a specialized biomechanical complex found between adjacent vertebral bodies, absorbs spinal column load and enables spinal mobility. A central nucleus pulposus (NP), an outer fibrous annulus, and upper and lower cartilage endplates constitute the IVD. Recently, mesenchymal stem cells were found in the nucleus pulposus (NP) and dubbed nucleus pulposus mesenchymal stem/progenitor cells (NPMSCs) [6]. What is more, NPMSCs showed similar biological characteristics to mesenchymal stem cells (MSCs), to include stem cell biomarker expression, self-renewal capabilities, and multilineage differentiation potential [4, 6–8]. NPMSCs cultured in an IVD mimicking microenvironments differentiate along a chondrogenic lineage, with a characteristic extracellular matrix secreted. Furthermore, under these conditions, NPMSCs showed a greater bioactivity when compared to adipose-derived MSCs [8–10], thus making these cells a potential intervertebral disc (IVD) regeneration therapy.

While stem cells possess therapeutic potential, one of the largest limiting factors is isolating seed cells. Presently, there are several ways to select and purify stem cells in vitro, such as an attachment-based culturing method [11, 12], fluorescent-based [13] or magnetic-based [14, 15] cell sorting, or density gradient centrifugation method [16, 17]. Among these methods, the best way to isolate NPMSCs remains unclear.

Recently, limiting dilution method, isolated stem cells from population depending on the clonogenic capacity of stem cells, was shown to easily select stem cells and aid in the maintenance of stem cell properties by controlling cell plating density [18, 19]. However, no study has reported if the limiting dilution method is suitable for isolating NPMSCs or an associated optimal plating density. Thus, this study examined rat NPMSCs cultured in three different plating densities and characterized differences in morphology, proliferative ability, migrative ability, stem cell biomarker expression (Sox2, Oct4, and Nanog), immunophenotyping, and multilineage differentiation potential (osteogenic, chondrogenic, and adipogenic).

## 2. Materials and Methods

### 2.1. Animals

The Lab Animal Center of Navy General Hospital supplied ten healthy male Sprague-Dawley rats (12 weeks old) to us. All studies were approved by the Institution Animal Care and Use Committee of Navy General Hospital.

### 2.2. Isolation and Culture of Rat NPMSCs

NPMSCs were obtained from the nucleus pulposus (NP) of rat caudal intervertebral disks (IVD) under aseptic conditions as previously described [8–10]. The NP was washed twice with phosphate-buffered saline (PBS; Hyclone, Logan, Utah, USA) and centrifuged at 1500 r/min for 5 min. The obtained NP was dissected into approximately 1 mm^3^ fragments and subsequently digested with 0.2% collagenase type II (Sigma-Aldrich, St. Louis, MO, USA) at 37°C for 6 h. The partially digested tissue, along with the emancipated cells, was suspended in low-glucose Dulbecco modified eagle medium (DMEM-LG; Solarbio Science & Technology Co., Ltd., Beijing, China) as an explant with 10% fetal bovine serum (FBS, Gibco BRL, Grand Island, NY, USA) supplemented with antibiotics in a moist atmosphere containing 5% CO_2_ at 37°C. The culture medium was changed every 3 days, with cells passaged at 70%–80% confluency. Harvested NPMSC-containing cell suspensions were filtered with a 40 *μ*m strainer (Falcon, USA) and then plated at low density (LD group; 5 cells/cm^2^), medium density (MD group; 100 cells/cm^2^), and high density (HD group; 10000 cells/cm^2^) in 25 cm^2^ cell culture flasks (Corning Incorporated, Corning, NY, USA). The LD cells were replated when the colonies became extremely large (at about 5% confluency); this way was also called limiting dilution method. The HD and MD cells were expanded until reaching approximately 80%–90% confluency. Next, all three groups were passaged at a density of 10000 cells/cm^2^ until 80%–90% confluency was attained.

### 2.3. Colony-Forming Ability Assay

NPMSC-containing cell suspensions from each group at passage 4 (P4) were plated in 6-well plates (Corning, USA) with DMEM-LG containing 10% FBS at a density of 200 cells/well, with the medium changed twice a week and cultures maintained for ten days. Samples were then fixed in 4% paraformaldehyde (Boster Biological Technology, Ltd., Wuhan, China), stained with 0.1% crystal violet solution (Solarbio Science & Technology Co., Ltd., Beijing, China) for 15 min, and washed twice in distilled water (DW). The numbers of colonies containing more than 30 cells were scored, with all samples examined in triplicate. The colony-forming rate was evaluated by dividing the number of colonies by the original number of adherent cells.

### 2.4. Transwell Migration Assay

The migratory abilities of cells from three groups were examined using a Transwell chamber (Corning, USA) with 5 *μ*m pore filter. The serum-free medium was used to suspend P4 cells for each group and cells sowed into the top chamber at a density of 10,000 cells/chamber, with complete medium (DMEM-LG containing 10% FBS) placed into the bottom chamber. After a 6.5 h incubation at 37°C in a moist atmosphere which contained 5% CO_2_, the cells that had not migrated from the top chamber were wiped off with a cotton swab. The cells that traversed the chamber and entered the film were steadied with 4% paraformaldehyde for fifteen min, dyed with 0.1% crystal violet solution (Solarbio Science & Technology Co., Ltd., China) for fifteen min, and washed twice with DW. Membranes were imaged under a light microscopy at 100x magnification in 3 randomly selected areas to calculate the average number of migrating cells.

### 2.5. CCK-8 Assay

Cell viability was evaluated using the Cell Counting Kit-8 (CCK-8, Dojindo Laboratories, Kumamoto, Japan) assay following the manufacturer's instructions. Briefly, the P4 cells from three groups were seeded at a density of 1000 cells/well in the 96-well plate (Corning, USA) in a volume of 100 *μ*l, and the NPMSCs were incubated overnight to allow for adherence. At 1, 3, 5, 7, and 9 days, CCK-8 reagents were put in each well and maintained for 2.5 h in a moist atmosphere at 37°C. Light absorbance was calculated at wavelength 450 nm with an automated microplate reader (Model 680, Bio-Rad Laboratories K.K., Tokyo, Japan) and experiment was examined in triplicate.

### 2.6. Cell Cycle Analysis

Cell cycle analysis was evaluated using a Cell Cycle and Apoptosis Analysis Kit (Beyotime Institute of Biotechnology, Haimen, China) after cells were washed twice in PBS. Briefly, 1 × 10^6^ cells at P4 from each density group were stabilized in glacial 75% ethanol overnight at 4°C. Each sample was dyed with propidium iodide (PI) staining buffer (0.5 ml buffer, 25 *μ*l propidium iodide, and 10 *μ*l RNase A) for 30 min at 37°C without light and analyzed using a FACSCalibur (BD Biosciences, USA) to detect fluorescence at 488 nm. Percentages of cells in G0/G1, G2/M, or S phases were assessed using the ModFit software (Verity Software House, Inc., Topsham, ME, USA) after applying the CellQuest software (BD Biosciences, USA) to obtain data. Experiment was examined in triplicate.

### 2.7. Multilineage Differentiation Assays

Osteogenic, adipogenic, and chondrogenic differentiation potential was assessed in cells from three density groups. To assess osteogenic and adipogenic potentials, cells were incubated in a six-well plate at a density of 2 × 10^4^ cells/cm^2^ and incubated overnight. The medium was then replaced with induction medium promoting either osteogenesis or adipogenesis (Cyagen Biosciences, Guangzhou, China). Cells were allowed to differentiate for 3 weeks, with the osteogenic induction media changed every 3 days and the adipogenic induction media changed following the manufacturer's instructions. Finally, the induction medium was discarded, and the cells were washed with PBS, fixed with 4% paraformaldehyde at 37°C for 30 min, and rinsed twice with PBS. Osteogenic differentiation was verified using alizarin red (Solarbio, China) staining and adipogenic differentiation was evaluated using oil red O (Solarbio, China) staining.

To evaluate chondrogenic differentiation, pellet cultures were generated. Briefly, cells at P3 from each density were resuspended in DMEM in a 15 mL polypropylene centrifuge tube. The gross of 5 × 10^5^ cells/tube were rinsed with incomplete chondrogenic medium (1% ITS supplement, 0.3% ascorbate, 0.1% sodium pyruvate, 0.1% proline, and 0.01% dexamethasone; Cyagen Biosciences, China) twice and centrifuged at 150*g* for five min. The cells were then suspended in 0.5 mL intact chondrogenic induction medium (incomplete chondrogenic medium + 1% TGF-*β*3; Cyagen Biosciences, China) and centrifuged at 150*g* for five min. The pellet was cultured at the bottom of the centrifuge tube in induction media at 37°C in 5% CO_2_ for 1-2 days and then the tube was softly flicked to guarantee the pellet floated freely in the medium. The medium was changed once every two days until reaching three weeks. At that time, the pellet was stabilized in 4% paraformaldehyde and inserted in paraffin. Alcian blue (Cyagen Biosciences, China) staining was applied to visualize the ECM and to assess sulfated proteoglycan formation. All experiments were performed in triplicate.

### 2.8. Semiquantitative Assay of Mineral Deposits, Adipogenic Capacity, and Alcian Blue Intensity

Following the induction of osteogenic differentiation, cells were dyed with alizarin red (Solarbio, China) for fifteen min and then rinsed with DW two times. The cells were then incubated with 600 *μ*l 10% cetylpyridinium chloride solution (Sinopharm Chemical Reagent Co., Ltd., Shanghai, China) at room temperature for five min and spectrophotometric (Bio-Rad Laboratories K.K., Japan) reading at OD_570_ nm was obtained. Following adipogenic differentiation, the cells were rinsed, fixed, stained with oil red O (Solarbio, China) for 30 min, and laid on a shaking table with isopropanol for 30 min at 30 revolutions/min. Lipid accumulation was then spectrophotometrically quantified at OD_490_ nm. Experiments were performed in triplicate, with mean values used to determine differentiation potential. Following chondrogenic differentiation, pellets were stained with alcian blue, with the stained areas visualized under a fluorescent microscope and quantified using ImageJ (NIH, Bethesda, MD, USA). All experiments were examined in triplicate.

### 2.9. Quantitative Real-Time PCR (qPCR) Analysis

Cells at P4 for each group were rinsed twice with glacial PBS and applied TRIzol reagent (Invitrogen Co., Carlsbad, CA, USA) to isolate total RNA. First, 1-microgramme total RNA was taken for cDNA synthesis using the Quantscript RT Kit (TIANGEN BIOTECH, China) at 37°C about 60 min following the manufacturer's instructions. The obtained cDNAs were then taken for quantitative real-time PCR (qPCR) using a SYBR® Premix Ex Taq™ (Tli RnaseH Plus; TaKaRa Bio, Otsu, Japan) in a Peltier thermal cycler (Bio-Rad Laboratories K.K., Tokyo, Japan) at an ultimate reaction volume of 20 *μ*l. After an initial denaturation for 30 s at 95°C, the DNA was amplified following 40 cycles of 10 s at 95°C and 30 s at 56°C, followed by a melting-curve analysis. Primer sequences for the detection of Sox2, Oct4, and Nanog were based on known mRNA coding sequences (Table 1). Experiments were performed in triplicate and expression levels of each target genes were normalized to *β*-actin mRNA levels. The cycle threshold (Ct) values for each gene were corrected using the mean ΔCt value and the 2 − ΔCt method was chosen to measure the relative expression of each target gene.

### 2.10. Immunophenotyping Analysis

Immunophenotype was analyzed using a flow cytometer with the following fluorescein isothiocyanate (FITC), allophycocyanin (APC), or phycoerythrin (PE) antibodies: CD29, CD44, CD90 (eBioscience, USA), CD34 (Abcam, Cambridge, MA, USA), and CD45 (BD Pharmingen, USA). Cells at p4 for each density were rinsed twice with glacial PBS and maintained in the dark for 30 min at 4°C with antibody. Finally, NPMSCs were rinsed three times with PBS and suspended with 500 *μ*l PBS for analysis on a FACSCalibur (BD Biosciences, USA). Experiments were performed in triplicate.

### 2.11. Statistical Analysis

The data expressed as the mean ± standard deviations (SD). One-way analysis of variance (ANOVA) followed by post hoc test (Dunnett's test) was performed to evaluate the differences among different plate densities, with *p* < 0.05 considered statistically significant.

## 3. Results

### 3.1. Changes in Morphology

**Figure 1:**
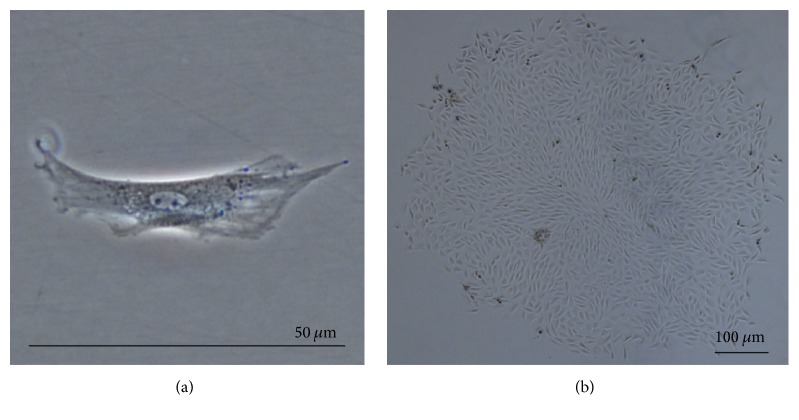
NPMSC LD group morphology at P1. (a) Single P1-LD NPMSCs displaying long spindle shaped morphology with short processes, abundant cytoplasm, and prominent nucleoli; magnification of 200x. (b) P1-LD NPMSC clusters displaying colony growth with homogeneous short spindle shaped morphology; magnification of 40x.

**Figure 2:**
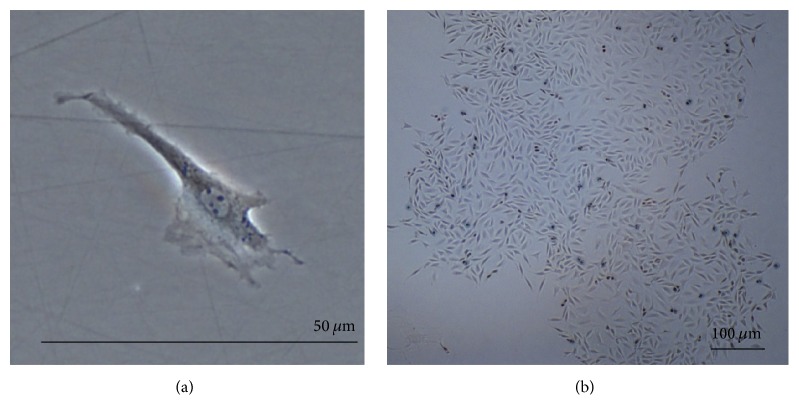
NPMSC MD group morphology at P1. (a) Single P1-MD NPMSCs displaying similar but shorter spindle shaped morphology relative to the LD group; magnification of 200x. (b) P1-MD NPMSC clusters displaying colony-like growth with a decreased percentage of short spindle shape cell and higher abundance of relatively round or polygonal shape cells; magnification of 40x.

**Figure 3:**
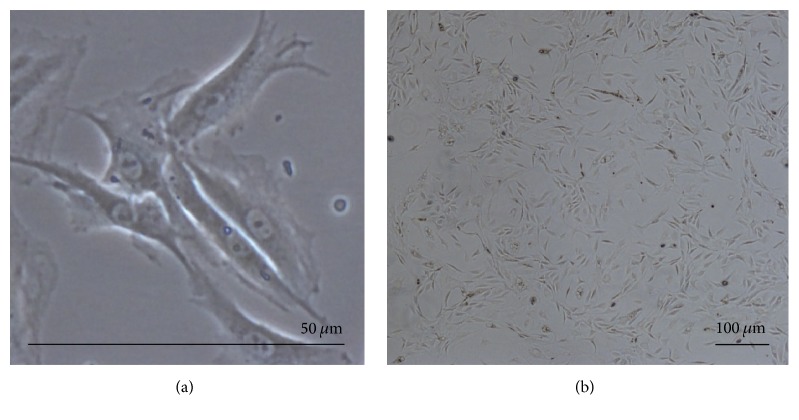
NPMSC HD group morphology at P1. (a) Single P1-HD NPMSCs displaying flattened, enlarged, and stellate morphology; magnification of 200x. (b) P1-HD NPMSC clusters displaying an irregular shape without colony growth; magnification of 40x.

**Figure 4:**
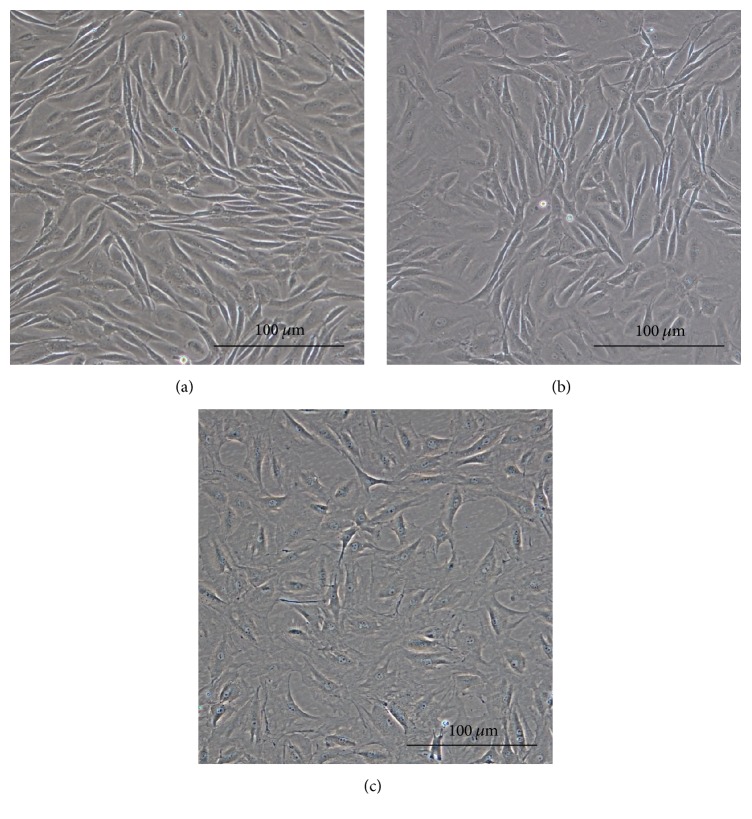
P2 cell cluster morphologies at different densities. (a) P2-LD NPMSC clusters showing a turbulence arrangement with an elongated spindle shape and high homogeneity. (b) P2-MD NPMSC clusters showing a short spindle shape with an inconspicuous turbulence arrangement. (c) P2-HD NPMSC clusters showing flattened and enlarged cells with low refractivity and higher heterogeneous shape. All images were taken at a magnification of 100x.

Harvested NPMSC-containing cell suspensions from each density were found to be plastic-adherent when incubated in standard culturing situations using tissue culture flasks. At passage 1 (P1), cells plated at LD and MD showed long, spindle shaped fibroblast-like morphology. In P1-LD cells, short, thick processes or pseudopodia were noted with an abundant cytoplasm containing large ovoid nuclei and prominent nucleoli (Figure 1(a)). Both P1-LD and P1-MD cells were single cell adherent to plastic (Figures 1(a) and 2(a)). P1-LD cells proliferated in a clone-like shape and had homogeneously sized cell populations with increased culture time (Figure 1(b)), while P1-MD cells had more relatively round or shorten shaped cells (Figure 2(b)). Furthermore, the P1-HD cells exhibited polygonal morphology (Figure 3(a)), with the percent of elongated spindle cells decreased and more cells becoming irregular with higher heterogeneity during culturing (Figure 3(b)). After passaging, most of the LD group cells showed a turbulence arrangement with slender and spindle shapes and high homogeneity (Figure 4(a)). In the MD and HD groups, a small percentage of spindle shaped cells were observed, with most being large polygonal or relatively round cells. These cells also showed flattened and enlarged morphology with low refractivity and higher heterogeneity (Figures 4(b) and 4(c)).

### 3.2. Improved Colony-Forming Capacity in LD and MD Culture

**Figure 5:**
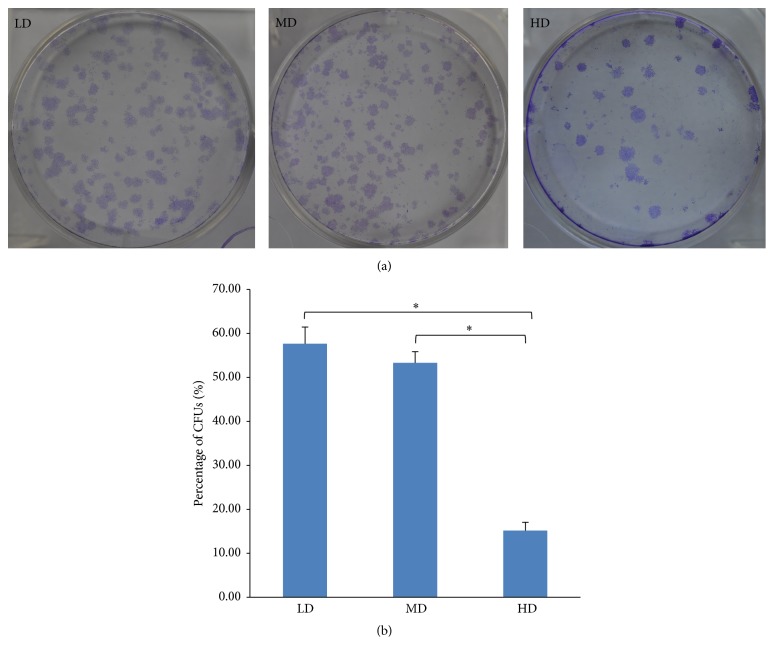
NPMSC colony-forming abilities. (a) Representative images of crystal violet staining of NPMSC LD, MD, and HD colonies after 10 days in culture, with a higher number of colony-forming units (CFUs) in the LD and MD groups. (b) Quantitative analysis showing significantly higher colony-forming efficiency in the LD and MD groups relative to the HD group. All samples examined in triplicate. The data are displayed as a mean ± SD, with ^*∗*^*p* < 0.05 deemed significant.

A colony-forming assay (Figure 5(a)) revealed that the LD and MD groups had higher numbers of larger colonies relative to the HD group. Further quantitative analysis (Figure 5(b)) also showed that the LD and MD groups had significantly higher colony-forming efficiency than the HD group (*p* < 0.05), while there was no difference between LD and MD group (*p* > 0.05). Overall, these findings showed a stronger colony-forming ability in cells culture in LD and MD.

### 3.3. Enhanced Migration Ability in LD Culture

**Figure 6:**
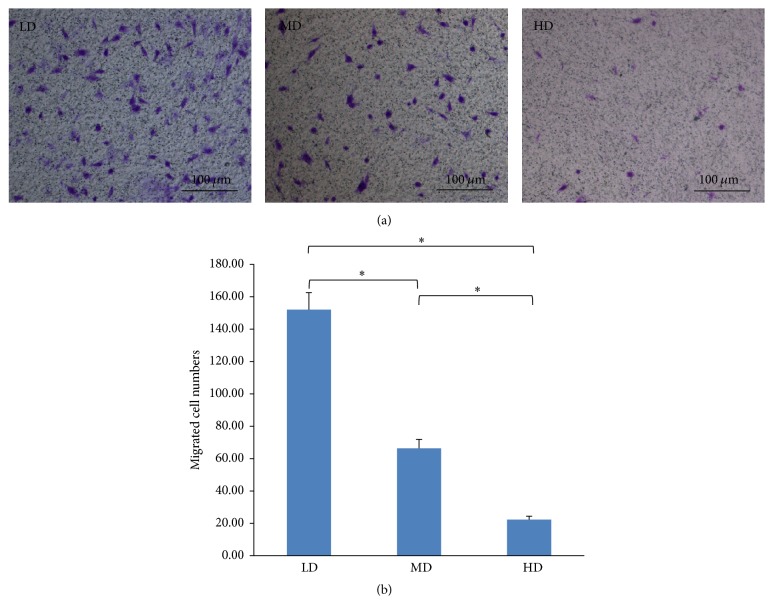
NPMSC migratory abilities in different culturing densities. (a) Migration was observed using a Transwell migration system with cells stained with crystal violet solution and observed under an inverted microscope (100x). (b) Migratory ability for each group was calculated as the average number of migrated cells within 3 examined fields and displayed as a mean ± SD. All samples examined in triplicate. The results showed that the number of migrating NPMSCs was higher in the LD group relative to the other groups at 6.5 h. ^*∗*^A significant difference of *p* < 0.05 relative to another group.

A Transwell migration assay was performed to evaluate the migratory abilities of NPMSCs from different culture density in vitro. At 6.5 h after incubation, the numbers of cells that migrated to the lower chamber in each group were determined as follows: (1) LD: 152.00 ± 10.54; (2) MD: 66.33 ± 5.51; and (3) HD: 22.33 ± 2.082 (Figure 6(a)). These results showed that the most pronounced migratory ability was present in the LD group (*p* < 0.05), followed by the MD and HD group (Figure 6(b)).

### 3.4. Changes in Cell Proliferation Capacity from Different Densities

**Figure 7:**
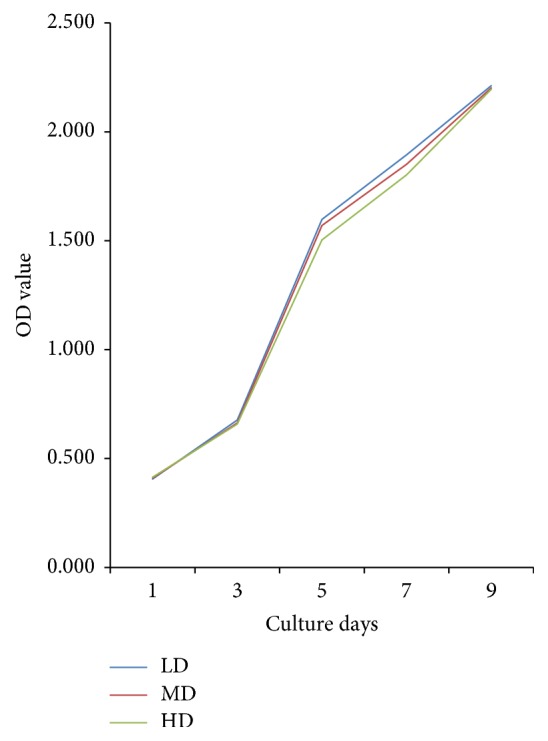
Determination of proliferative capacity of NPMSCs at different densities using the CCK-8 assay. While the OD value of LD group was slightly higher than that of the MD and HD groups at the logarithmic phase, no significant difference was noted between the groups. All samples examined in triplicate.

Regarding the proliferation capacity, all cells from each group exhibited similar growth tendencies. The OD values rose slowly during the initial 3 days after plating, thus indicating a slow growth period. A sharp increase occurred from days 3 to 7, with a plateau noted from days 7 to 9. While a slightly higher OD value was noted in the LD group relative to the other groups, the differences were not significant (*p* > 0.05, Figure 7).

### 3.5. Cell Cycle Changes under Different Culture Densities

**Figure 8:**
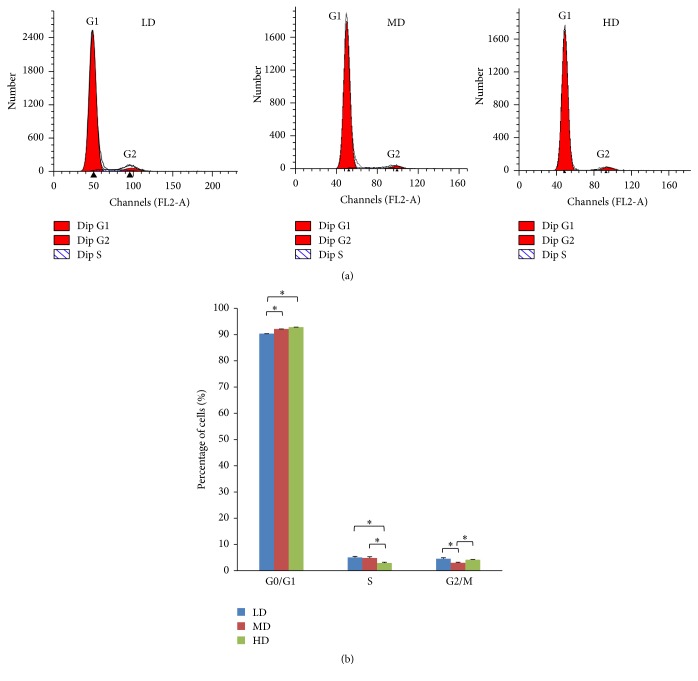
Effects of culturing density on cell cycle distributions in NPMSCs. (a) Representative cell cycle graphs of NPMSCs cultured at different densities. The percentage of NPMSCs in each phase of the cell cycle was determined via flow cytometry. (b) While the majority of cells from each group were mainly in the G0/G1 phase (>90%), a decreased G0/G1 phase arrest and increased S phase entry were observed in the LD and MD groups. All samples examined in triplicate. The data are displayed as a mean ± SD, with ^*∗*^*p* < 0.05 deemed significant.

The effect of density on cell cycle distribution was evaluated in cells of each group. The average percentage of cells in either G2/M, S, or G0/G1 phases was examined and showed average percentages of 92.90%, 2.93%, and 4.18% for P4-HD cell population, 92.17%, 4.85%, and 2.98% for P4-MD population, and 90.36%, 5.09%, and 4.55% for P4-LD population, respectively (Figure 8(a)). Comparing to the other two groups, the highest percentage of S phase cells was noted in the P4-LD population (*p* < 0.05), with no significant difference noticed between the LD and MD groups (Figure 8(b)).

### 3.6. Changes in Multilineage Differentiation Potential under Different Culture Densities

#### 3.6.1. Osteogenic Differentiation

**Figure 9:**
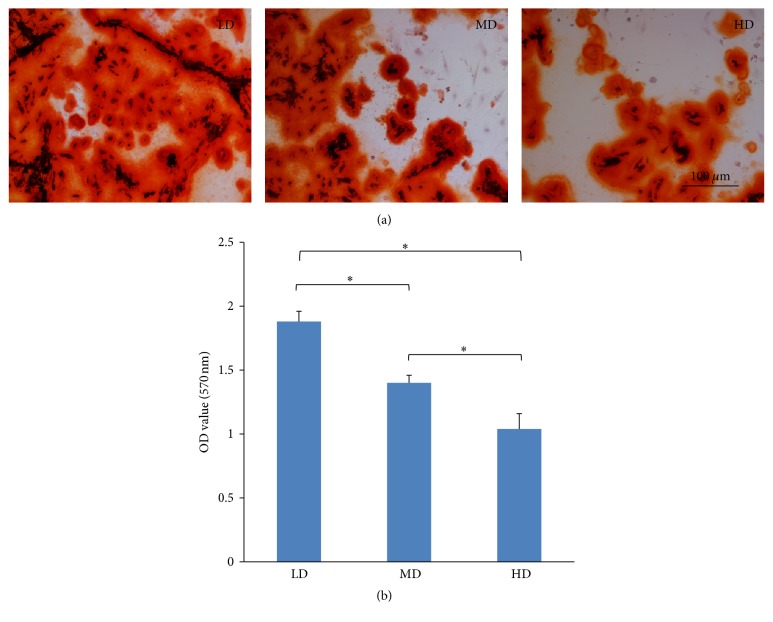
Osteogenic potentials of NPMSCs from three density groups. (a) Osteogenic differentiation was determined using alizarin red staining (100x), with LD NPMSCs observed to form bigger and more intensely stained mineralized nodules. (b) A semiquantitative assay of mineral deposition, with the highest levels of deposition seen in the LD > MD > HD groups. All samples examined in triplicate. The data are displayed as means ± SD, with ^*∗*^*p* < 0.05 deemed significant.

All the NPMSC-containing cell suspensions of each group, irrespective of culturing density, were shown to be positive for alizarin red 3 weeks after differentiation induction (Figure 9(a)). Morphologically, the LD cell population displayed larger and more intensely stained mineralized nodules, thus indicating more extensive calcium deposition. Semiquantitative osteogenic capacity analysis was performed on dye quantification at OD_570_ nm (Figure 9(b)). The highest OD value was seen in the LD group (1.877 ± 0.082), followed by the MD and HD groups (1.403 ± 0.059 and 1.040 ± 0.123), with all comparisons between groups deemed significant (*p* < 0.05). Overall, these consequents suggest that NPMSCs from the LD group had a higher osteogenic potential relative to the MD and HD groups.

#### 3.6.2. Adipogenic Differentiation

**Figure 10:**
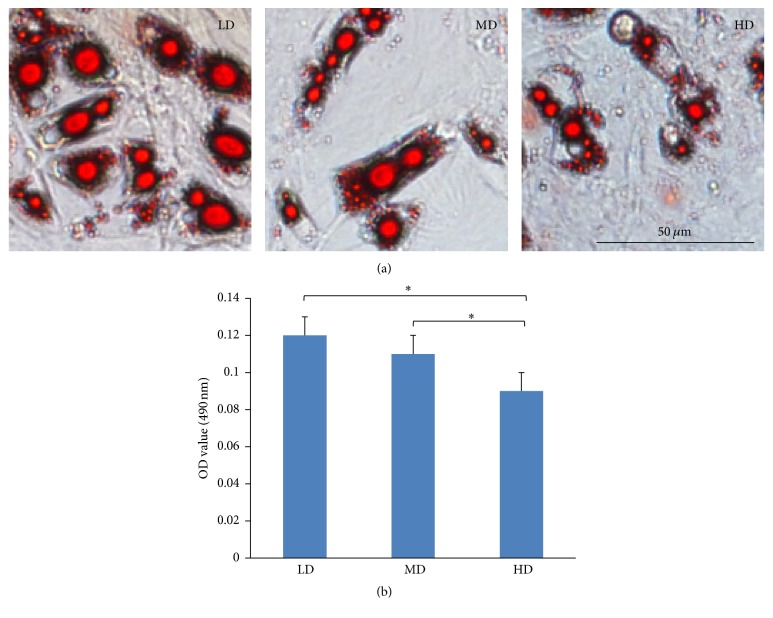
Adipogenic potentials of NPMSCs from the three density groups. (a) Adipogenic differentiation was histologically determined using red O staining (100x). All cells exhibited lipid formation, but cells from the LD group displayed larger and more abundant lipid vacuoles. (b) A semiquantitative assay of lipid formation, with the highest levels seen in the LD > MD > HD groups. All samples examined in triplicate. The data are displayed as a mean ± SD, with ^*∗*^*p* < 0.05 deemed significant.

Three weeks after adipogenic induction, cell suspensions of each group showed the presence of lipid-rich vesicles dyed with oil red O. The highest levels of lipid accumulation were noted in the LD group relative to the other groups (Figure 10(a)). To determine the semiquantitative adipogenic capacities, staining was quantified at OD_490_ nm and showed the highest lipid accumulation levels in the LD group (0.121 ± 0.006), followed by the MD and HD groups (0.112 ± 0.007 versus 0.088 ± 0.007; Figure 10(b)), which suggest that NPMSCs from the LD group had a higher adipogenic potential relative to the MD and HD groups. Significant differences were noted between each of the groups (*p* < 0.05), with the exception of between the LD and MD groups.

#### 3.6.3. Chondrogenic Differentiation

**Figure 11:**
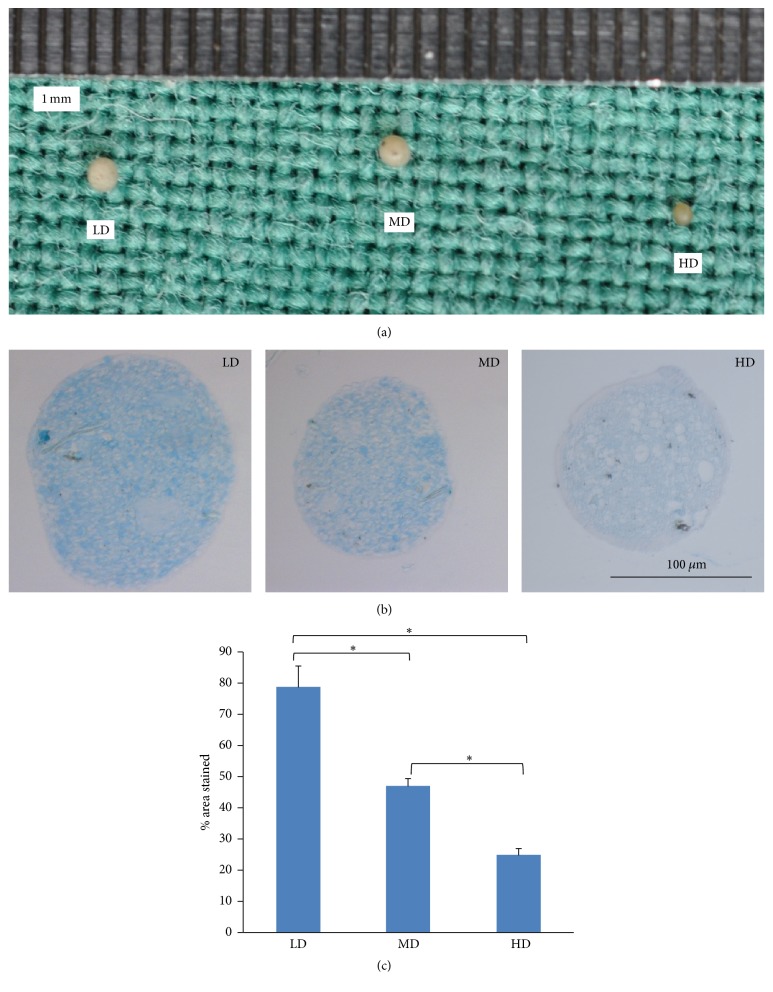
Chondrogenic potentials of NPMSCs from the three density groups. (a) During chondrogenesis, an increase in the pellet size was noted. (b) Chondrogenic differentiation was evaluated following alcian blue staining (200x). All cell pellets exhibited a degree of alcian blue staining, but the most abundant and intense staining was seen in the LD group. (c) A semiquantitative assay of proteoglycan deposits that further confirmed the histological appearances. All samples examined in triplicate. The data are displayed as a mean ± SD, with ^*∗*^*p* < 0.05 deemed significant.

To evaluate chondrogenic differentiation potential, pellet cultures were formed. During chondrogenesis, an increase in the pellet size was noted, with the largest size increase noted in the LD group (Figure 11(a)). Histological examination showed positive alcian blue staining for all of the groups, with the most intense staining noted in the LD group (Figure 11(b)). A semiquantitative assay determined that the percentage of sulfated proteoglycan was significantly higher in the LD group relative to the other two groups (*p* < 0.05; Figure 11(c)), thus indicating that the NPMSCs of LD group had the greatest chondrogenic differentiation potential.

### 3.7. Stem Cell Biomarker Expressional Changes under Different Culturing Densities

**Figure 12:**
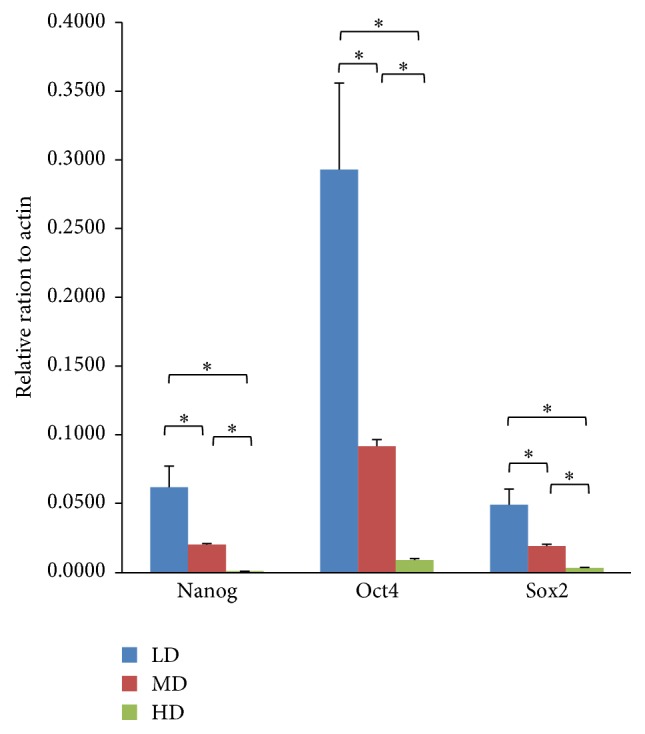
Quantitative real-time PCR (qPCR) analysis of stem cell biomarkers. P4-LD NPMSCs showed the highest expression levels of Nanog, Oct4, and Sox2, followed by the MD and HD groups. Each experiment was performed in triplicate (*n* = 3), with a significant difference (^*∗*^*p* < 0.05) noted between each group comparison.

The expression levels of common stem cell biomarkers, to include Nanog, Oct4, and Sox2, were examined in passage 4 cells. The P4-LD cell population showed highest expression levels of these biomarkers (*p* < 0.05) relative to the other two groups. These findings indicate that the expression levels of these pluripotent markers are downregulated as culture density increased (Figure 12).

### 3.8. Features of Immunophenotyping

**Figure 13:**
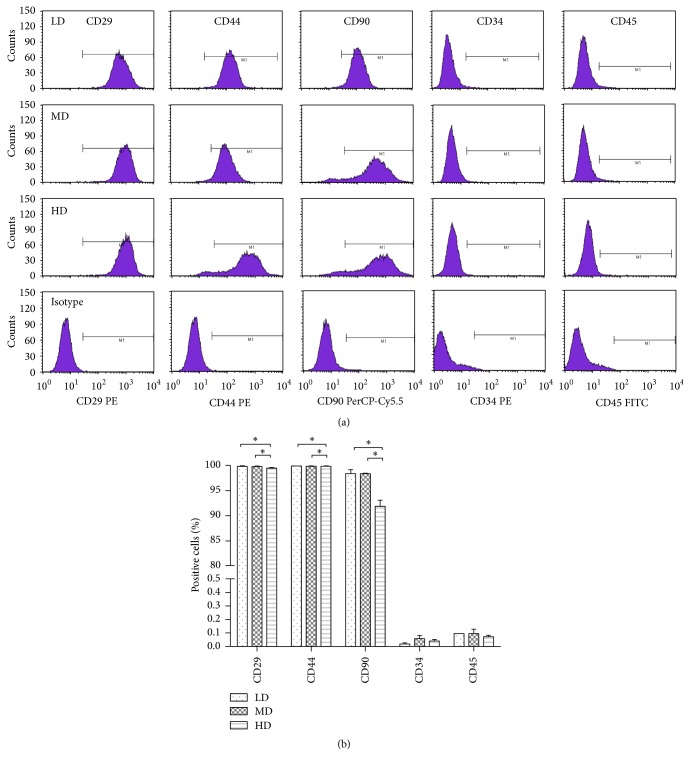
Immunophenotype analysis of NPMSCs from the three density groups. (a) Immunophenotype graphs from the three density groups. (b) All NPMSCs from the three groups were positive for CD29, CD44, and CD90 and negative for CD34 and CD45. The LD group exhibited the highest percentage of cells expressing positive cell markers, followed by the MD and HD groups. All samples examined in triplicate. Data are presented as a mean ± SD, with ^*∗*^*p* < 0.05 deemed significant.

The antigenic phenotype of NPMSC-containing cell suspensions from each group was characterized by flow cytometric analysis (Figure 13(a)), with cells found to be positive for CD29, CD44, and CD90. The LD group was found to have the highest percentage of markers positive cells relative to the other groups (*p* < 0.05), followed by the MD and HD groups. All cells from each group were negative for the hematopoietic stem cell marker CD34 and the pan-hematopoietic marker CD45 (Figure 13(b)).

## 4. Discussion

Nowadays, MSCs implantation has become a promising method for the treatment of IVDD. With stem cells possessing therapeutic potential, to investigate the relationship between the function and state endogenous stem cells derived from NP with the pathogenic mechanisms contributing to IVDD, the key point here is the isolation of the stem cells. Recently, researchers had found and harvested the MSCs from the human NP [6, 7] and Sprague-Dawley rat [8–10], dubbed nucleus pulposus mesenchymal stem/progenitor cells (NPMSCs). All these articles isolated the NPMSCs from the NP through adherent culture and successive passaging method without special handling. However, Friedenstein et al. [20] found that not all adherent mesenchymal cells isolated from bone marrow are regarded as MSCs; only those cells exhibiting colony-forming ability could be the candidate for MSCs. Although there is no similar data for nucleus pulposus cells, the data obtained with bone marrow mesenchymal cells are instructive: it is highly probable that similarly to bone marrow mesenchymal stem cells (BMMSCs), only a fraction of nucleus pulposus cells, which exhibit colony-forming ability, corresponds to definition of a multipotent stem cell. It is a biological minimum to define a mesenchymal stem cell (as multipotent, high proliferative potential cell).

Therefore, utilizing the clonogenic capacity of stem cells, the limiting dilution method was chosen to isolate the periodontal ligament stem cells (PDLSCs) from periodontal ligament [21], the clonogenic mesenchymal progenitor cells from umbilical cord blood [22], gingival-derived mesenchymal stem cells (GMSCs) from gingival connective tissue [23], and tendon-derived stem cells (TDSCs) from the patellar tendons of rat [24]. However, if the limiting dilution method is suitable for isolating NPMSCs or an associated optimal plating density remains unanswered, in this study, to our knowledge, the limiting dilution method was used in isolating NPMSCs with good biological activities and stem cell characteristics for the first time, and this method was cheap, simple, and easy operation. The effect of plating density was also examined with regard to biological characteristics and tissue engineering applications. Our results demonstrated that while NPMSC-containing cell suspensions from three plating densities shared similar morphologies and proliferative abilities, the LD cell population had higher homogeneity and more of small, spindle shaped morphology.

One of the most significant aspects of tissue engineering is an ability to isolate a large quantity of seed cells with high plasticity. Previous studies have suggested that utilizing a limiting dilution method when isolating MSCs under LD condition can yield a cell population with a higher proliferative capacity and activity [25, 26]. One possible reason for this outcome may be that the LD culture selects small MSCs (cell size < 10 *μ*m) [27] that exhibit a higher colony-forming ability and thus display a rapid expansion in culture [27, 28]. In the present study, NPMSCs in the LD group (5 cells/cm^2^) displayed a higher colony-forming activity and enhanced migratory ability, which may also have been attributed to a smaller cell size. As previously described, none but tumor or stem cells can survive and proliferate in the clone shape and colony-forming ability is one of the important characteristics of stem cells [20, 29, 30]. Since in the LD population the incidence of C-FUF is 57.67 ± 3.79%, in MD population 53.33 ± 2.57%, and in HD population 15.17 ± 1.89%, there might be higher proportion of stem cells in LD group compared to the other two groups.

Furthermore, cell cycle analysis showed a higher percentage of cells that had entered S phase in the LD group, with G0/G1 phase arrest prevented (*p* < 0.05). As the CCK-8 assay showed no significant difference in viability of cells at different densities though OD value of LD group was slightly higher than other two groups, this might be due to lack of the number of samples. The finding further suggested that the limiting dilution method might select NPMSCs from adherent nucleus pulposus cells (NPCs) with higher cell viability and proliferative abilities. The benefits of the limiting dilution method may then be further enhanced when seeding at a lower density, possibly due to adequate surface being provided for extensive replication and more available nutrients per cell.

In agreement with previous studies showing that NPMSCs express MSC surface biomarkers [6], this study also found the cells to be CD29 (integrin *β*1), CD44 (hyaluronate receptor), and CD90 positive, while being CD34 and CD45 negative (hematopoietic stem cells marker). Furthermore, the highest percentage of stem cell biomarkers positive cells was found in the LD group, thus indicating that a larger fraction of NPMSCs were isolated following the limiting dilution method. Additionally, the multilineage differentiation potentials of the isolated NPMSC from three groups were also examined. Following semiquantitative analysis of osteogenic and adipogenic differentiation, the NPMSCs of LD group exhibited the highest differentiation potential for these two lineages when compared with the other groups. The chondrogenic differentiation potential was examined using a pellet culture, with the pellet size monitored and the degree of alcian blue staining observed. These results showed that the pellet size increased as seeding density decreased, with the highest percent area of alcian blue staining noted in the LD group. Collectively, these results showed that the highest multilineage differentiation capacity was achieved under LD condition, with the capacities ranked as follows: LD > MD > HD, thus indicating that LD culture maintained or promoted NPMSCs plasticity.

Key stem cell biomarkers, to include Oct4, Sox2, and Nanog [27], were also examined, with the highest expression levels seen in NPMSCs of the LD group and gradually decreasing with increased plating density. Previous studies have shown that Oct4 is associated with the maintenance of self-renewal ability in somatic cells, while Sox2 and Nanog play crucial roles in colony-forming capability, proliferation, and multidifferentiation ability [27, 31]. Thus, the upregulation of these pluripotent markers under LD conditions may be related to an improved cell proliferation and multilineage differentiation capability. Despite the strength of these findings, the samples used herein were not of human origin and therefore potential differences within the rat IVD biochemical composition may be present. Furthermore, the mechanisms and related signal pathways associated with plating density-related functional changes in NPMSCs require further examination.

Previous studies showed a similar method in isolating NP stem/progenitor cells. Study from Sakai et al. [32] isolated NP stem cells by methylcellulose semisolid medium and study from Erwin et al. [33] suspended cells in neural basal A medium under hypoxic conditions (3.5% O_2_) to collect free-floating colonies. These two approaches were based on 3D culture. According to the studies [32, 33], there were straightforwardly less than 1 × 10^2^ cells after being cultured for 10 days in 3D culture. In our study, we chose 2D culture and gained more than 1 × 10^3^ cells during the same time. We speculated that the suitable oxygen concentration and 2D plastic adhesion might due to higher proliferative capacity and cell count.

In conclusion, this paper is the first to report on the effects of utilizing the limiting dilution method in the isolation of rat NPMSCs in vitro to our knowledge. By means of quantitative and qualitative comparisons, the effects of the limiting dilution method on various NPMSC biological characteristics and a determination of a plating density able to promote cell activity and plasticity were established. The findings presented herein will aid in further laying a foundation for the use of NPMSCs in tissue engineering or cell therapy. In our future studies, we will investigate the mechanisms impacted by using the limiting dilution method and whether this method may be applied in tissue engineering and regenerative medicine.

## Figures and Tables

**Table 1:** Primer sequences for qRT-PCR.

Gene name	Forward sequence	Reverse sequence
*β*-Actin	AgATCCTgACCgAgCgTggC	CCAgggAggAAgAggATgCg
Nanog	TCTggggACCTACCTCTT	CTTggTgAggACCTTgTTC
Sox2	gCACCgCTACgACgTCAg	gCCTCggACTTgACCACA
Oct4	ggACACCTggCTTCAgACTT	TCCACAgAACTCgTATgCTg
